# Editorial: Leadership and management in organizations: Perspectives from SMEs and MNCs

**DOI:** 10.3389/fpsyg.2023.1156727

**Published:** 2023-03-14

**Authors:** Saeed Siyal

**Affiliations:** School of Economics and Management, Beijing University of Chemical Technology, Beijing, China

**Keywords:** leadership, management, training and development, MNCs, SMEs, ethical and social perspectives, moral dimensions, contemporary issues

## Introduction

Leadership and management are fundamental aspects for the smooth performance, progress, and growth of any organization with respect to its nature and the way it operates (Siyal et al., 2021a). Every multinational, national, SME, and corporate sector needs effective management and leadership. This allows them to perform smoothly and produce significant output, which leads to growth and development. It is believed that the best leadership and management make remarkable contributions to the growth of institutions and yield remarkable outcomes (Siyal and Peng, 2018; Kouzes and Posner, 2023). This is due to the increasing recognition of and demand for effective leaders and managers by the corporate sectors, MNCs, and SMEs that are aiming to lead the global market. For this, they need qualified, trained, and committed leaders and managers who can efficiently and effectively lead the team, resources, and market. It is evident that several organizations acknowledge the need for effective leadership and efficient management, but they are still uncertain about the proper management, leadership style, and behaviors that are most effective for the growth and development of the corporate sector, MNCs, and SMEs, along with the development of their human resources (Kelly and Hearld, 2020; Siyal et al., 2021b). Considering all these aspects and conflicting results from the past, the role of leadership and management in the smooth operationalization, growth, and development of the corporate sector, MNC, and SMEs remains unresolved.

## Summary

This Research Topic focuses on leadership and management in organizations in diverse workplace settings including MNCs and SMEs. The call for papers was published between February 2022 and August 2022, during which the COVID-19 pandemic continued to impact some regions but not others. Scholars and practitioners were invited to submit research articles and brief reports pertaining to leadership and management in organizations, including corporations and SMEs, in the field of organizational psychology to the Frontiers in Psychology journal. In response to this call for papers, a huge number of academicians and practitioners submitted their research. Out of a total of 81 submissions, 18 were accepted and published under this theme. The Research Topic includes studies from diverse cultural and industrial settings, such as academia and industries including MNCs and SMEs, from across the globe. The businesses covered in this Research Topic are enterprises, manufacturers, SMEs, MNCs, educational institutes, logistics SMEs, and government sectors. The submitting authors were from different countries, including the Republic of Korea, China, Pakistan, Saudi Arabia, the United Arab Emirates (Abu Dhabi), Oman, Qatar, Serbia, and Poland. The authors came up with new research approaches and methodologies, contributing to the theory and practice in this important emerging research domain of leadership and management.

Xu et al. investigated whether and how differential leadership in SMEs influences subordinate knowledge hiding. They analyzed the underlying mechanisms of chain-mediator–job insecurity and territorial consciousness and the boundary condition–leadership performance expectation. The results indicated that differential leadership plays a potential role in promoting subordinate knowledge hiding through the serial intervening mechanism of job insecurity and territorial consciousness in SMEs. This study contributes to the existing academic literature by empirically analyzing the under-investigated correlation between differential leadership and subordinate knowledge hiding in SMEs and by exploring the underlying mechanisms and boundary condition.

Ding et al. examined the link between an employee's professional identity and their success *via* the mediating role of critical thinking. They also examined the interaction of an employee's professional commitment and a leader's motivational language by critically analyzing employee success. This study was conducted on Chinese MNCs by use of a time-lagged study design. The results show a positive relation between an employee's professional identity and their success. Furthermore, the critical analysis mediated the link between professional identity and employee success.

Jun et al. examined the impact of supervisors' authentic leadership styles on the turnover of their subordinates in multiple organizations in the Republic of Korea. Their findings generalized the effects of leadership on turnover across different research contexts. Furthermore, they proposed a new mechanism and tested the mediation and moderation of the supervisor-perceived support and organizational identification, which reduces the turnover rate and help organizations to retain their best talents.

## Conclusion

Leadership and management in organizations are crucial elements in ensuring the success of both MNCs and SMEs. While MNCs may have more resources and a more formal structure, SMEs often have a more flexible and agile approach to leadership and management. Both MNCs and SMEs can benefit from effective leadership and management practices such as clear communication, setting achievable goals, fostering a positive work culture, and continuously adapting to changing market conditions. Ultimately, the key to success in both types of organizations is having leaders and managers who are able to inspire and motivate their teams to achieve their goals.

In this regard, this Research Topic has introduced this novel research work by scholars and researchers from around the globe, with a focus on leadership and management perspectives and their role in organizations. Thus, the selected Research Topic is very important for the business, industry, management, academic, and economic value of practitioners and academic institutions at all levels, as well as those with country-wide and international offices. This Research Topic has contributed through novel approaches and provide suggestions for the managers and leaders of corporate sectors, MNCs and SMEs, academicians, academic institutions, social scientists, students, policymakers, government and non-government agencies, and other related stakeholders. Similarly, the findings in this Research Topic have suggested that investigating the impact of leadership and management in organizations could influence future research, and further studies could compare the effectiveness of leadership and management in MNCs and SMEs.

## Author contributions

The author confirms being the sole contributor of this work and has approved it for publication.
